# Abnormal nociception and opiate sensitivity of STOP null mice exhibiting elevated levels of the endogenous alkaloid morphine

**DOI:** 10.1186/1744-8069-6-96

**Published:** 2010-12-20

**Authors:** Alexandre Charlet, Arnaud H Muller, Alexis Laux, Véronique Kemmel, Annie Schweitzer, Jean-Christophe Deloulme, Denise Stuber, François Delalande, Enrica Bianchi, Alain Van Dorsselaer, Dominique Aunis, Annie Andrieux, Pierrick Poisbeau, Yannick Goumon

**Affiliations:** 1 Institut des Neurosciences Cellulaires et Intégratives, Centre National de la Recherche Scientifique et Université de Strasbourg, Strasbourg, F-67084, France; 2 Inserm, U575, Physiopathologie du Système Nerveux, Strasbourg, F-67084, France; 3 EA-4438, Faculté de médecine, Université de Strasbourg, Strasbourg, F-67085, France; 4 Inserm, U836, Institut des Neurosciences; CEA/iRTSV/GPC, Université Joseph Fourrier, Grenoble, F-38042, France; 5 Laboratoire de spectrométrie de masse BioOrganique, IPHC-DSA, ULP, CNRS UMR7178, Strasbourg, F-67087, France; 6 Department of Neuroscience, University of Siena, Siena, Italy

## Abstract

**Background-:**

Mice deficient for the stable tubule only peptide (STOP) display altered dopaminergic neurotransmission associated with severe behavioural defects including disorganized locomotor activity. Endogenous morphine, which is present in nervous tissues and synthesized from dopamine, may contribute to these behavioral alterations since it is thought to play a role in normal and pathological neurotransmission.

**Results-:**

In this study, we showed that STOP null brain structures, including cortex, hippocampus, cerebellum and spinal cord, contain high endogenous morphine amounts. The presence of elevated levels of morphine was associated with the presence of a higher density of mu opioid receptor with a higher affinity for morphine in STOP null brains. Interestingly, STOP null mice exhibited significantly lower nociceptive thresholds to thermal and mechanical stimulations. They also had abnormal behavioural responses to the administration of exogenous morphine and naloxone. Low dose of morphine (1 mg/kg, i.p.) produced a significant mechanical antinociception in STOP null mice whereas it has no effect on wild-type mice. High concentration of naloxone (1 mg/kg) was pronociceptive for both mice strain, a lower concentration (0.1 mg/kg) was found to increase the mean mechanical nociceptive threshold only in the case of STOP null mice.

**Conclusions-:**

Together, our data show that STOP null mice displayed elevated levels of endogenous morphine, as well as an increase of morphine receptor affinity and density in brain. This was correlated with hypernociception and impaired pharmacological sensitivity to mu opioid receptor ligands.

## Background

Stable tubule-only polypeptides (STOP) are a family of calmodulin binding and regulated microtubule associated proteins (MAPs), encoded by a single gene in mouse (Mtap6) [1,2] and human (MAP6) [3]. These proteins have been firstly identified as microtubule stabilizer [1,2,4] and play an important role in neuron morphology, function [5,6] and migration [7,8]. STOP proteins are also able to interact with actin cytoskeleton [9], with membranous compartments through palmitoylation events [10] and are found in synaptosomal fractions [11] indicating potential synaptic functions. Accordingly, STOP null mice display alterations of integrated brain functions compatible with some symptoms of schizophrenia including neuroleptic-sensitive behavioural abnormalities [11-13]. This mice model exhibit increased basal locomotor activity during the dark phase of the light/dark cycle, purposeless and disorganized activity, severe social withdrawal and nurturing defects [11,14]. In particularly, STOP null mice have synaptic defects in the hippocampus well correlated with a depletion of glutamatergic vesicle resulting in a defective long-term potentiation (LTP) and long-term depression (LTD) in the CA1 hippocampal area [11,15]. Hypersensitivity to acute stressful situations, hyperlocomotion after amphetamine administration and dopamine hyper-reactivity in the limbic system have also been described [12]. With respect to the latter observation, electrically-evoked dopamine release is selectively increased in the nucleus accumbens of STOP null mice, whereas basal extracellular dopamine levels are not changed in the striatum or in the nucleus accumbens [11,12].

At the transcriptional level, STOP invalidation is associated with a decrease of synaptophysin, VGlut1 (vesicular glutamate transporter-1), and spinophilin mRNAs in the hippocampus and in the cerebellum [16]. Interestingly, spinophilin, a dendritic spine-enriched scaffold protein, is a modulator of opiate effects [17]. Thus, spinophilin invalidation reduces sensitivity to the analgesic effects of morphine but also the early development of tolerance. Spinophilin appears to be associated with the mu opioid receptor (MOR) in striatum and modulates MOR both at the signaling and endocytosis levels. A stimulation of MOR by morphine promotes a suppression of MOR responsiveness [17].

Recent results suggested that endogenous morphine (eM), whose structure is identical to that of morphine isolated from poppies (for review see [18-20]), might represent an interesting novel neuromodulator of brain function. Although still under investigation, eM presence and synthesis has been characterized in numerous mammalian cells [21-23] and tissues including brain [24-26]. Morphine is particularly present in the hippocampus, striatum, cortex, hypothalamus, cerebellum, and in key structures of the nociceptive system such as the midbrain periaqueductal gray matter, nucleus raphe magnus, rostroventral medulla complex and amygdala [23,27-31]. In mammals, the biosynthesis of eM derives at least from dopamine [32-34]. Thus, eM biosynthesis and release were recently shown in the SH-SY5Y human neuronal catecholamine-producing cell line [23,32,35]. Endogenous morphine is likely involved in different stress-modulating or pain-modulating mechanisms *via *binding to MORs which are expressed by numerous cell types (*e.g.*, neurons and immune cells) [21,36-38]. Stimulation of these receptors leads to various effects, including analgesia but also modulation of hormone synthesis and secretion (*e.g.*, CRH), as well as immunosupression [21,39]. Endogenous morphine, which is present in nervous tissues and synthesized from dopamine, may contribute to these behavioral alterations since it is thought to play a role in normal and pathological neurotransmission. To date, the functional role of eM in the brain remains to be elucidated and only few data are available regarding the presence of morphine in cerebral areas with regards to its possible consequences on brain function.

The present study shows for the first time that STOP null mice exhibit elevated levels of eM in different macrostructures of the brain. The physiological consequences of such high brain content of eM have been investigated with regards to the potential associated changes in morphine receptor (density and affinity) and basal nociception.

## Results

### Endogenous levels of morphine in WT and STOP null mice brains and sera

**Figure 1:**
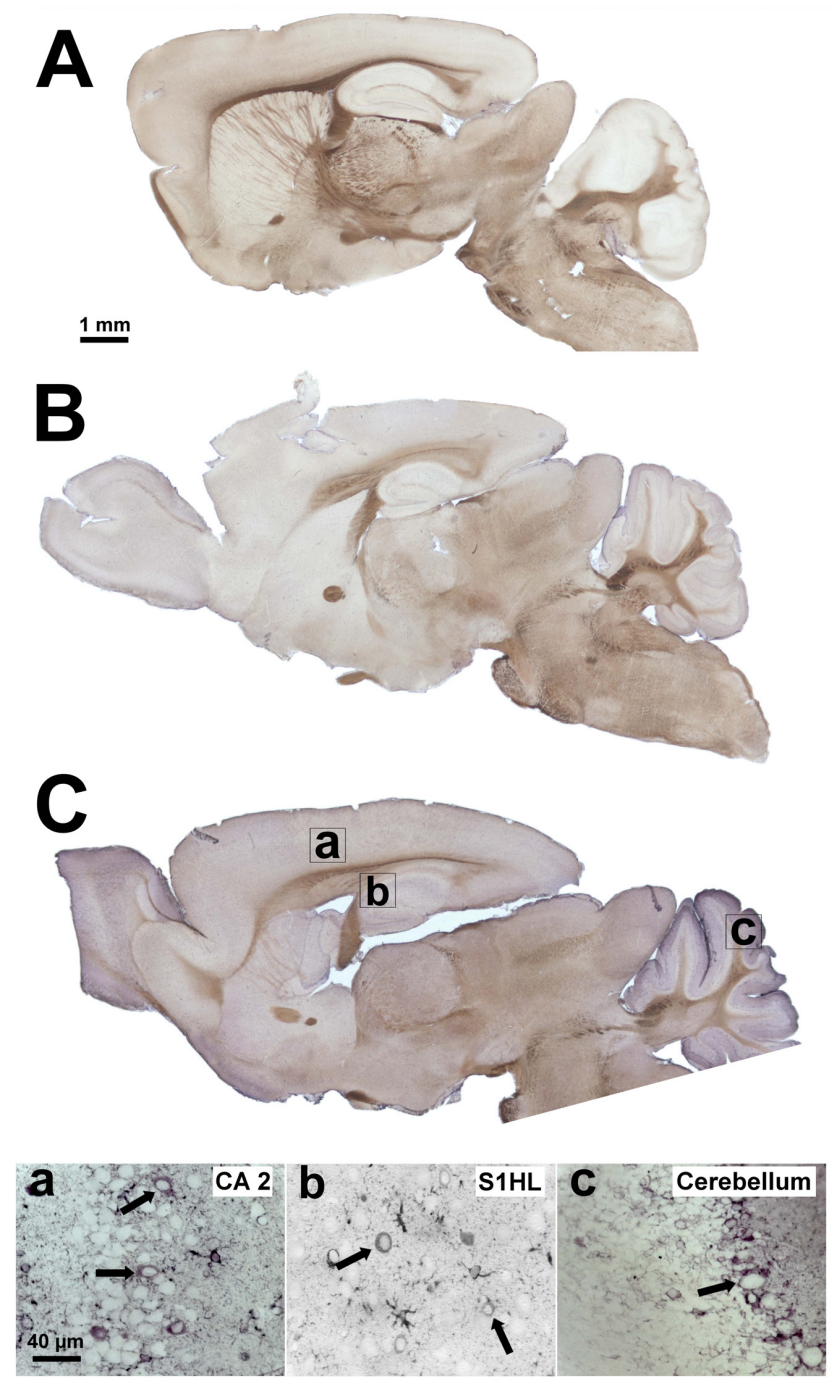
**Immunodetection of morphine-like compounds in the brain of WT and STOP animals**. **A**. Control experiment using anti-morphine immunoadsorbed mouse monoclonal antibody (same incubation time as in B and C). **B**. Immunodetection of morphine-like molecules in WT mouse brain. Sagittal slices were incubated with a mouse monoclonal anti-morphine antibody and visualized with an HRP-conjugated donkey anti-mouse IgG. **C**. Immunodetection of morphine-like compounds in STOP null brain. Boxes indicated the areas of interest. **Ca-c **panels correspond to the areas defined by the boxes. Arrows indicate eM immunoreactive neurons. S1HL, hind limb primary somatosensory (S1HL) cortex.

**Figure 2:**
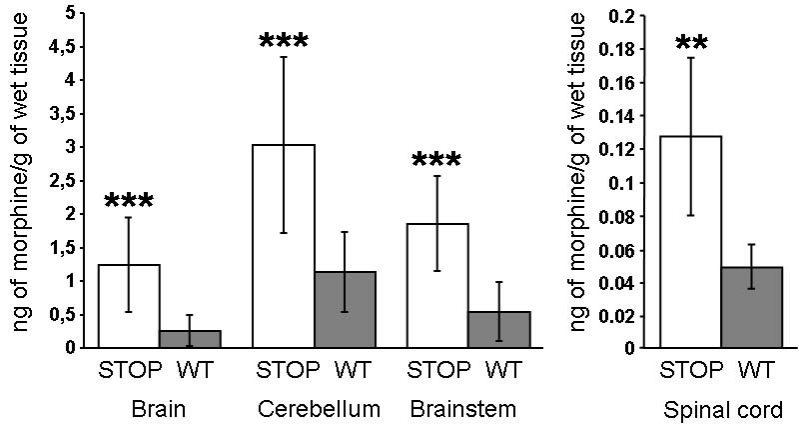
**Quantification of the morphine contents in different mouse brain areas of WT and STOP null mice using a morphine-specific ELISA**. Amounts of endogenous morphine (ng) present in the cerebellum, brainstem and the remaining brain tissue, per gram of wet tissue. The values correspond to the morphine amount determined for STOP null (n = 10 animals; white bars) and WT (n = 13 animals; grey bars) mice in different cerebral areas as indicated. For spinal cord, the values correspond to the morphine amount determined for STOP null (n = 9 animals; white bars) and WT (n = 9 animals; grey bars) mice. Amounts of eM in the STOP null and WT groups were found statistically different (mean +/- SD, Mann-Whitney test using Bonferroni correction; **: p < 0.01; ***: p < 0,001).

The presence and localization of eM and its derivatives was examined in the encephalon of WT and STOP null mice by immunohistochemistry. The 6D6 antibody has previously been validated for the detection of morphine, morphine-6-glucuronide (M6G), morphine-3-glucuronide (M3G) and codeine [21]. A stronger immunoreactivity for morphine-like compounds is observed for the brain of STOP null mice compared to wild type mice (Figure 1B and 1C) suggesting the presence of a higher concentration of endogenous morphine. This immunoreactivity increase was global and no particular areas were labeled in STOP null mice as compared to WT animals. No immunoreactivity for eM or derivatives was found in control experiments using morphine, M6G, M3G and codeine-immunoadsorbed antibody (Figure 1A and data not shown). In STOP null animals, morphine-like immunolabel was found in different brain areas implicated in nociception. Thus, morphine-like immunoreactivity was found in neurons of the hippocampus CA2 area (arrows Figure 1Ca), hind limb primary somatosensory (S1HL) cortex (arrows Figure 1Cb) and the cerebellum (immunolabel around Purkinje cell bodies; arrow Figure 1Cc). Quantifications of morphine levels in cerebellum (Figure 2), brainstem and remaining parts of the brain (*i.e.*, brain without cerebellum and brainstem), as well as in spinal cord, were performed using a morphine-specific ELISA kit (see methods). Quantification of eM present in WT animals shows the presence of morphine in brain (0.25 ± 0.23 ng/g of tissue; n = 13), cerebellum (1.13 ± 0.59 ng/g of tissue; n = 13), brainstem (0.54 ± 0.44 ng/g of tissue; n = 13) and spinal cord (0.05 ± 0.01 ng/g of tissue; n = 9; Figure 2). In STOP null mice, a statistically higher amount of morphine per gram of tissue was found in the brain (1.24 ± 0.69 ng/g of tissue; 483 ± 273% compared to WT; p < 0.001; n = 10; mean +/- SD, Mann-Whitney test using Bonferroni correction), cerebellum (3.03 ± 1.31 ng/g of tissue, 268 ± 116% compared to WT; p < 0.001; n = 10), brainstem (1.85 ± 0.7 ng/g of tissue, 341 ± 130% compared to WT; p < 0.001; n = 10) and spinal cord (0.13 ± 0.04 ng/g of tissue, 257 ± 93% compared to WT; p < 0.01; n = 9; Figure 2).

In order to determine if the endogenous morphine level in the blood is also altered, a quantification using a morphine-specific ELISA was performed on the serum of STOP null (n = 8) and WT (n = 8) mice. Morphine was undetectable in the serum of the two strains of mice (*i.e.*, under the detection limits; data not shown).

Together, these data indicate that STOP null mice cerebral areas contain a significantly higher amount of eM compared to WT animals.

**Figure 3:**
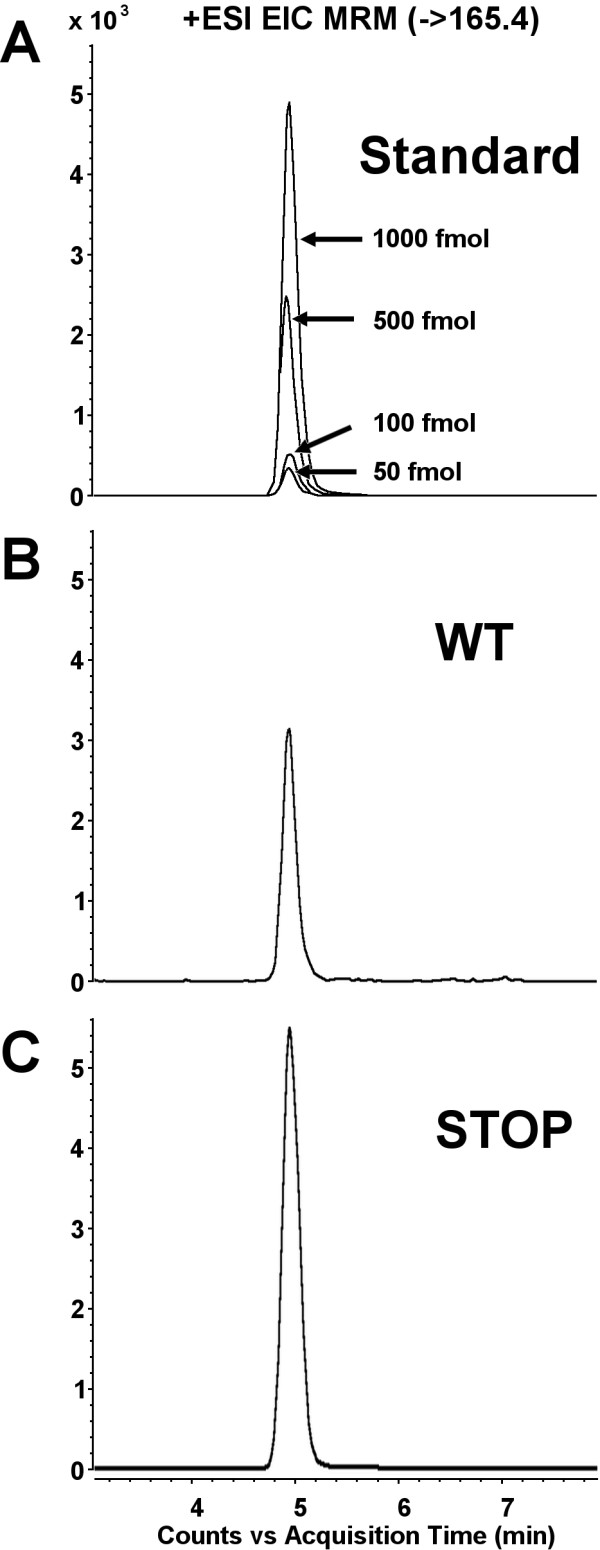
**Characterization of morphine in mouse brain extracts**. Morphine qualification was performed in selected reaction monitoring (SRM) mode with the following transitions: m/z 286.2 → 165.1 (see also additional file 1). SRM trace of the specific transition for morphine in **A**: standard samples of morphine (50, 100, 500 and 1000 fmol); **B**: WT brain animals and **C**: STOP null brain animals.

To confirm the presence of morphine in brain extracts of STOP and WT mice, LC-MS-MS analyses were performed in SRM mode. The specific transition m/z 286.2 → 165.1 was used to qualify the presence of morphine. The results obtained unambiguously confirm the presence of morphine in mouse brain of both WT and STOP null mice and are summarized in Figure 3 (3A for standard morphine, 3B for WT mice and 3C for STOP null animals).

### Morphine binding in STOP null and WT mice

**Figure 4:**
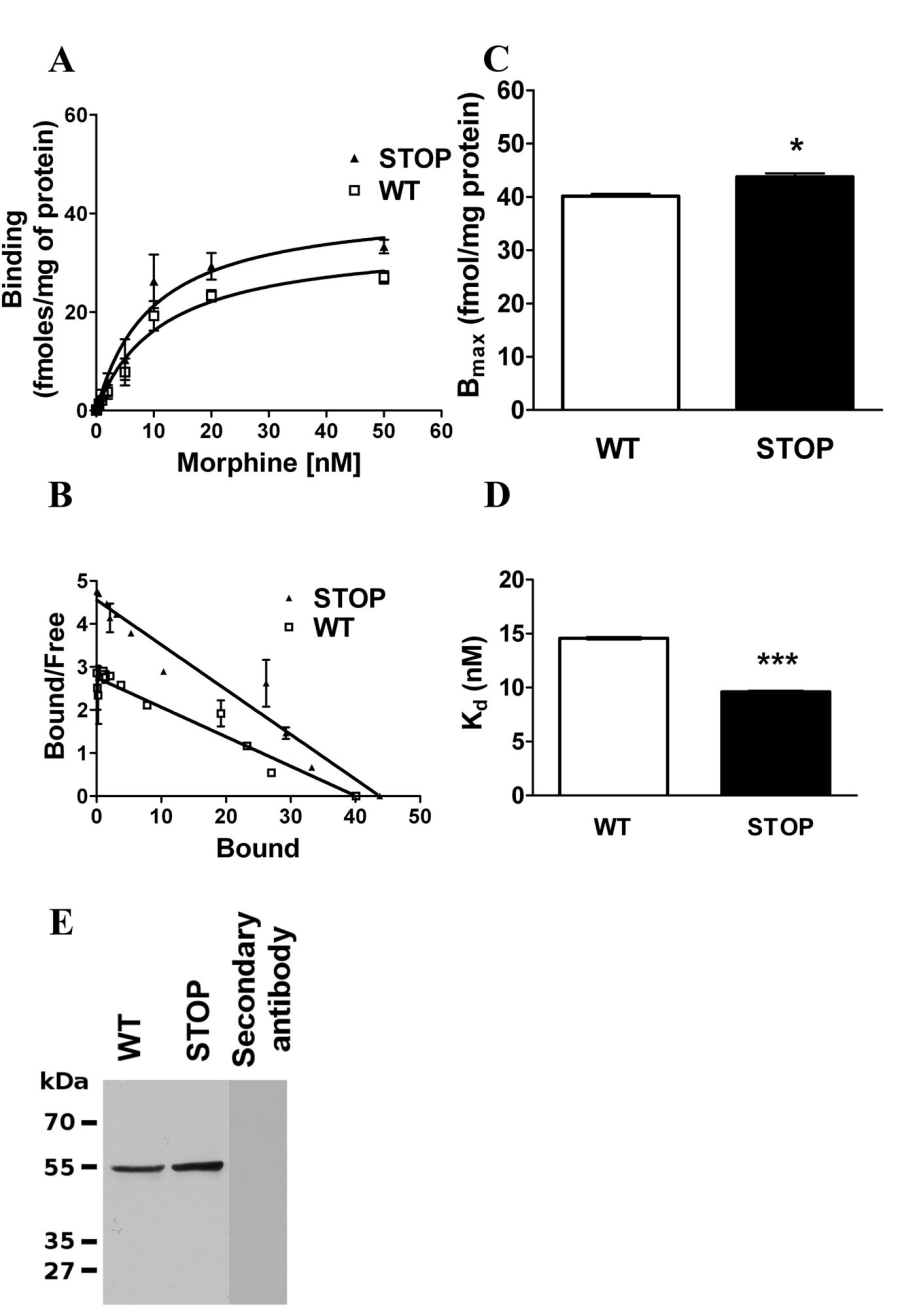
**[^3^H]-Morphine binding in WT and STOP null mice**. **A **: Saturation analysis of [^3^H]-morphine binding to the MORs in wild-type and STOP null mice. Data are presented as means ± S.E.M. n = 3. **B **: Transformation of the data by linear regression_Scatchard plots. Data are presented as means ± S.E.M. n = 3. **C **: Comparison of the *B*_max _values of MORs in wild-type and STOP null mice. **D **: Comparison of the *K*_d _(C) values of MORs in wild-type and STOP null mice. Data are presented as means ± S.E.M. n = 3, Statistical significance between STOP null versus WT mice is indicated as follow: * p < 0.05, ** p < 0.01, *** p < 0.001 by Student *t*-test. **E **: Western blot analysis showing MOR immunolabel present in 50 μg of brain proteins of wild-type and STOP null mice (n = 3). No cross reactivity was found for the secondary antibody used.

In order to determine specific *K*_d _and *B*_max _values of MORs, a saturation analysis was performed on a pool of brain membrane of STOP null (5 brains pooled for 3 independent experiments) or WT mice (7 brains pooled for 3 independent experiments) using [^3^H]-morphine as radioligand (Figure 4A). Analysis of saturation binding curves yield an accurate estimation of *K*_d _and *B*_max _values (Figure 4A and 4B). In STOP null mice, the *K*_d _value of MORs was 9.6 ± 0.2 nM (mean ± SD, n = 3; Figure 4D). This value was found to be statistically lower by 35% compared to wild-type mice (14.6 ± 0.2 nM, mean ± SD, n = 3, *P *< 0.0001, Student *t*-test). The statistical analysis to compare variance (F-test; F = 1.0, DFn = 2.0, Dfd = 2 and p = 1) assumes that variance aren't significantly different. The *B*_max _of morphine opioid receptors determined for STOP null mice (43.8 ± 2.5 fmol/mg of protein, mean ± SD, n = 3; Figure 4C) was higher (around 9%) than in wild-type mice (40.1 ± 3.5 fmol/mg of protein, mean ± SD, n = 3, *P = *0.0104, *t*-test), showing the presence of a higher density of MORs in the brain of the knock-down model. The statistical analysis to compare variance (F-test; F = 1.96, DFn = 29, Dfd = 29 and p = 0.075) assumes that variance aren't significantly different. The linear-Scatchard plot regression analysis leads to the same results.

Western blot analysis was performed in order to confirm that MOR protein expression is increased in STOP null mice compared to wild type animals. In both extracts, immunoreactivity was observed as a band at 55 kDa which is consistent with the expected molecular weight of the mu opioid receptor (Figure 4E). MOR protein amount was found to increased by 12% in STOP extracts (average n = 3) compared to wild type animals. A control experiment using the secondary antibody alone showed that no cross reactivity exists (Figure 4E).

Together, our data suggest that STOP deficiency lead to a significant increase of MORs affinity and density in whole brain compared to WT mice.

### Mechanical and thermal nociception in WT and STOP null mice

Mechanical and thermal nociception of STOP null versus WT animals were investigated with von Frey and dynamic hot/cold plate assays, respectively.

**Figure 5:**
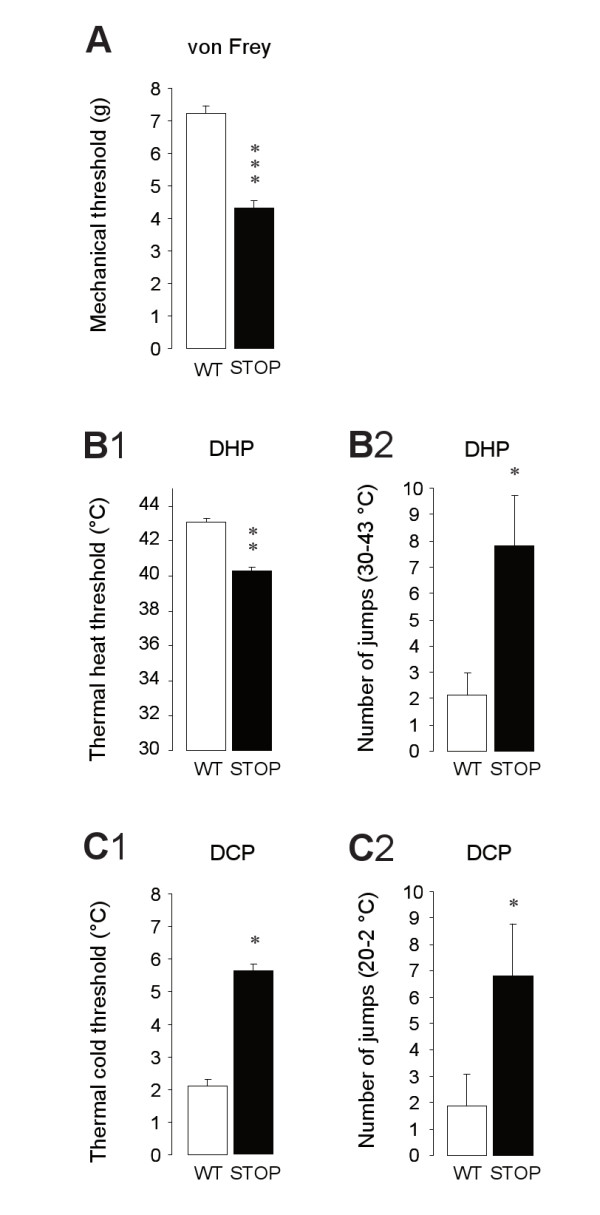
**Thermal Nociceptive thresholds and behaviors of WT and STOP null mice**. **A**. Mechanical von Frey thresholds of WT (n = 17) and STOP null (n = 16) mice. **B**. Thermal hot nociceptive thresholds (B1) and behaviors (B2: total number of jumps between 30 and 43°C) for WT (n = 16) and STOP null mice (n = 16). **C**. Thermal cold nociceptive thresholds (C1) and behaviors (C2: total number of jumps between 20 and 0°C) for WT (n = 10) and STOP null mice (n = 18). Statistical significance between STOP null versus WT mice is indicated as follow: * p < 0.05, ** p < 0.01, *** p < 0.001.

STOP null mice display a significantly lower mechanical nociceptive threshold compared to WT (Figure 5A). The mean threshold was of 7.24 ± 0.22 g and of 4.31 ± 0.24 g for the WT littermates (n = 17) and STOP null mice (n = 16; p < 0.001), respectively. Thermal nociception was evaluated with hot (Figure 5B) and cold temperature stimulations (Figure 5C) using dynamic ramps. In the dynamic hot plate test (DHP: 1°C/minute from 30°C to 45°C), we found that STOP null mice (n = 16) had a significantly lower nociceptive hot threshold (Figure 5B1) with mean value of 40.25 ± 0.70°C compared to WT mice (43.06 ± 0.47°C, n = 17; p < 0.01). In good agreement with the result obtained in the hot range, a significantly bigger mean cold threshold was obtained for STOP null mice (5.61 ± 0.91°C; n = 18) in comparison to WT mice (2.10 ± 0.71°C; n = 10; p < 0.05) when the mice were exposed to dynamic cold plate test (DCP: 1°C/min; from 20°C to 0°C; Figure 5C1). It is interesting to note here that only 29.41% (10 mice out of 34) of WT mice but 68.75% (18 mice out of 32) of STOP null mice were producing jumps during the DCP test. While counting the number of jumps in the hot (from 30 to 42°C; Figure 5B2) and cold range (from 20 to 2°C; Figure 5C2), we found that STOP null mice were systematically hypernociceptive (heat: WT, 2.13 ± 0.85, STOP, 7.81 ± 1.91, p < 0.05; cold: WT, 1.85 ± 1.22, STOP, 6.81 ± 1.97, p < 0.05). In summary, STOP null mice displayed a significant higher sensibility to thermal and mechanical stimuli than WT.

### Morphine and naloxone modulation of mechanical nociception

**Figure 6:**
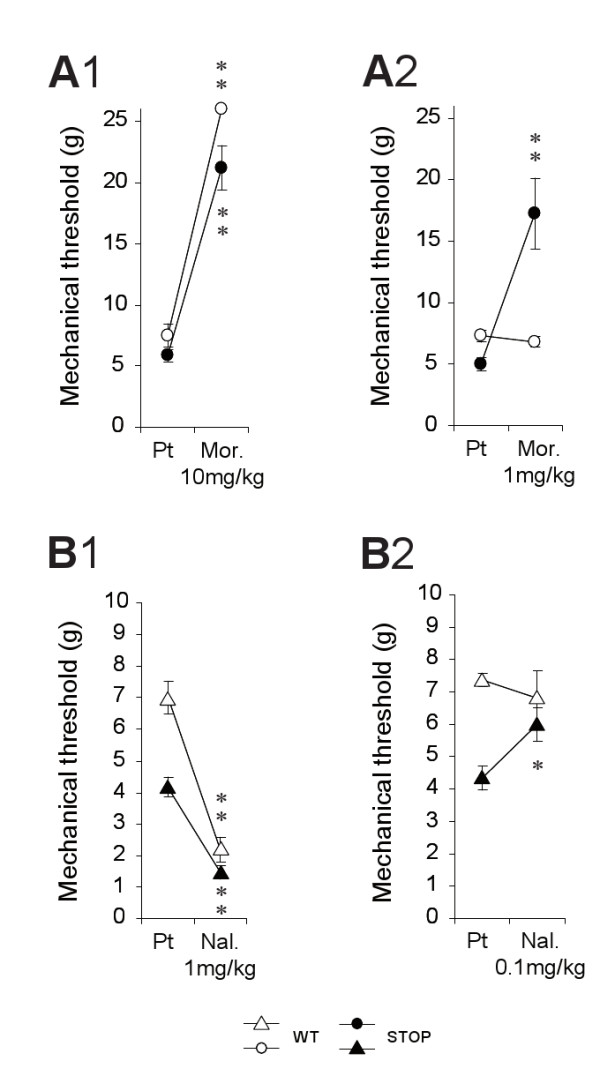
**Effects of morphine and naloxone on mechanical nociceptive threshold**. **A**. Effects of a single subcutaneous morphine injection, at 10 mg/kg (A1) and 1 mg/kg (A2), on mechanical von Frey threshold for WT (n = 6) and STOP null mice (n = 6). **B**. Effects of a single subcutaneous naloxone injection, at 1 mg/kg (B1) and 0.1 mg/kg (B2), on mechanical von Frey threshold of WT (n = 6) and STOP null (n = 6) mice. Statistical significance is indicated as follow: * p < 0.05, ** p < 0.01.

To evaluate the antinociceptive properties of morphine on mechanical nociceptive threshold (von Frey filaments), mice received a single subcutaneous injection of morphine at a high (10 mg/kg) or low (1 mg/kg) concentration (Figure 6A). As expected, administration of high dose (10 mg/kg) of morphine (Figure 6A1) induced a significant increase of the mechanical threshold in WT mice (from 7.50 ± 0.92 g to the cut-off value of 26 ± 0 g; n = 6, p < 0.01) and STOP null mice (from 5.83 ± 0.48 g to 21.17 ± 1.83 g; n = 6, p < 0.01). Interestingly, injection of low dose (1 mg/kg) of morphine (Figure 6A2) did not change the mean mechanical threshold of WT animals (from 7.50 ± 0.50 g to 7.0 ± 0.45 g; n = 6, p > 0.05) whereas it significantly increased the value of STOP null mice (from 5.00 ± 0.58 to 17.25 ± 2.90; n = 6, p < 0.01). Saline subcutaneous injections (vehicle of morphine) were without effect in both WT and STOP null mice (data not shown; n = 6 per group).

In addition to morphine, we also characterized the effects of high (1 mg/kg) and lower dose (0.1 mg/kg) of naloxone, an antagonist of opioid receptors, on mechanical nociceptive threshold (Figure 6B). Following the injection of high dose of naloxone (Figure 6B1), the mean threshold of the WT mice was decreased from 7.00 ± 0.52 g to 2.17 ± 0.40 g (n = 6; p < 0.01) and from 4.17 ± 0.31 g to 1.45 ± 0.20 g (n = 6, p < 0.01) in WT and STOP null mice, respectively. A slightly different situation was observed while testing lower dose of naloxone (Figure 6B2). In this case, no change was observed in the mechanical threshold of WT mice (from 7.36 ± 0.20 g to 6.79 ± 0.88 g; n = 11, p > 0.05), whereas in STOP null mice, naloxone significantly increase the nociceptive threshold from 4.33 ± 0.37 g to 6.00 ± 0.53 g (n = 9, p < 0.05).

## Discussion

The present study first indicated that the brain of STOP null mice express high level of endogenous morphine (eM) in comparison to WT littermates. MORs present in the brains of STOP null mice were expressed at a higher density and displayed a higher affinity for morphine compared to WT mice. With respect to possible consequences linked to altered eM brain content and MORs alterations, we found that STOP null mice were hypersensitive to nociceptive stimuli [40] and showed unusual nociceptive and motor responses following morphine receptor ligand injections. Indeed, subcutaneous injections of low doses of morphine (1 mg/kg) were ineffective in WT mice but produced a significant antinociception and reduced locomotion in STOP null mice. In line with this result but more unexpected, a low dose of the antagonist naloxone was antinociceptive in STOP null mice whereas it remained without effect in WT. Altogether, these data lead to the hypothesis that STOP null mice display an altered brain eM content and MOR pharmacology which might be responsible for the abnormal behaviours seen after morphine receptor ligand administration or acute nociceptive stimuli.

### Elevated levels of endogenous morphine in the brain of STOP null mice

Endogenous morphine has been previously described in bovine, rat, monkey and dog brains (for review: [18,20]), as well as in human neuroblastoma SH-SY5Y cells [23,32]. We also found that eM is particularly strongly expressed in the sensory motor cortex, hippocampus, cerebellum and spinal cord. These areas express MORs [41-44] thus suggesting that eM might be an important homeostatic modulator of their respective functions. In sharp contrast with the brain of WT animals, we found that STOP null mice display higher levels of eM in general. Since the biosynthesis of eM requires the presence of dopamine [20,32-34], elevated levels of morphine may result from an over production of dopamine. To support this hypothesis, STOP null mice have been shown to display dopamine hyper-reactivity in the limbic dopaminergic system and increased dopamine release in the nucleus accumbens following electrical stimulation of the medial forebrain bundle [11,12]. Alternatively, a possible uptake of circulating eM (or of its precursors) by nerve cells and specific brain structures could also explain why some dopamine-free brain regions express eM. Further investigations will be required to fully explain why higher eM levels are found in the brain and spinal cord of STOP null mice but this observation might be linked to their atypical response to stress [12].

### Elevated affinity and density of MORs in the brain of STOP null mice

Our present study showed that STOP deficiency is associated with an increase in the affinity and the density of MORs in mice brain. These data, together with the high levels of endogenous morphine found in STOP null mice, raise several questions regarding the possible cellular mechanisms that could be involved. Usually, upregulation of receptors may results from an increased receptor synthesis and decreased receptor degradation. The increased affinity of opioid receptors has been previously shown to result from a decrease of phosphorylation, oligomerization and allosterism of opioid receptors [45]. Because STOP proteins directly alter spinophilin function and interaction with actin cytoskeleton [16,17], this suggests the elevated morphine receptor affinity to possibly result from an impaired receptor targeting at the membrane and/or recycling. However, those changes in morphine concentration could affect the affinity and the density of morphine receptors [46,47]. We can hypothesize that the occurrence of a higher affinity and density of MORs in STOP null mice brain might explain the effect of low morphine concentration (1 mg/kg) compared to WT mice. Further experiments will be required to identify the molecular/cellular mechanisms involved in the case of STOP null mice.

### Endogenous morphine and basal hypernociception of STOP null mice

At this time, the physiological consequences of eM presence at various concentrations in bovine, rat, rodent, monkey and dog brains remains to be better understood [18,20,21]. Among the few evidence published in the literature indicating that eM might affect brain functions, intracerebroventricular injections of an antibody raised against the alkaloid morphine (*i.e. *immunoneutralization) have suggested a role for eM in weakening memory processes under stress conditions [48]. This result on memory performance shows that stressful conditions are clearly involving the endogenous morphine system. Because we found higher eM content in the brain and spinal cord of STOP null mice and since the function of nociceptive central nervous system structures might be affected [27-31], we characterized their nociception in comparison to their WT littermates. STOP null mice do not display spontaneous nociceptive responses but they were hypersensitive to acute mechanical and thermal nociceptive stimuli. Our finding is in apparent contrast with the thermal hot hypernociception seen after immunoneutralization of eM in mice [28]. The reduction of paw withdrawal latency in the hot-plate test at 52°C likely indicated that, in basal condition, an eM-dependent antinociceptive tonic control was present. It is interesting to note at this point that treatment with various doses and durations of exogenous morphine or derivatives has also ambivalent properties being sometime hypoalgesic or hyperalgesic [49-51]. It suggests that abnormal eM levels might be the substrate for this hypernociception in STOP null mice. We cannot fully explain at this stage why STOP null mice are hyper-responsive to thermal and mechanical nociceptive stimuli, but we can propose that the high levels of eM seen in the spinal cord and brain of STOP null mice profoundly alters the pharmacology and the function of opioid receptors. Alternatively, the presence of high eM may be a compensatory mechanism to preserve a minimal opioidergic antinociception despite major defects in the trafficking and/or properties of opioid receptors resulting from the STOP deletion. In good agreement with this proposal, STOP invalidation was shown to decrease the mRNA levels of a dendritic spine-enriched scaffold protein named spinophilin [16]. Further experiments will be required to confirm this hypothesis, but the behavioral consequences of exogenous morphine and naloxone administration strongly support this proposal.

### Effects of opioid receptor ligands on nociception

Two different concentrations of morphine have been tested on nociception. In STOP null mice, it seems that over-expression of eM in their brain is not associated with decrease in morphine-sensitivity. In the contrary, subcutaneous injection of a low dose of morphine (1 mg/kg, Figure 6A2) induces no analgesia in WT whereas it produces a strong analgesia in STOP null mice. It appears then that STOP null mice are either hypersensitive to exogenous morphine or, alternatively, may benefit from the existing basal eM concentrations to reach an analgesic efficacy after low s.c. injection of morphine. In line with these abnormal opioid responses to exogenous opiates, injection of the mu-receptor antagonist naloxone, at 0.1 mg/kg, induce a slight but significant analgesia in STOP null mice but was without effect in WT animals. Several articles report that low doses of naloxone are able to enhance morphine analgesia [52-54]. In our model, it is surprising to see that the presence of high amount of eM in STOP null mice whole encephalon and spinal cord is accompanied by a basal hyper-nociception for all modalities tested compared to WT. Such results may eventually be compared to chronic morphine treatments, that are described to induce tolerance and hyperalgesia [52-54].

### Physiological consequences and potential clinical significance of eM increase

In STOP null mice eM amounts present in brain increase and both thermal and mechanical differences compared to control animals may be related to MOR pharmacology abnormality. Our data suggest that an increase of eM amount may directly affect the pharmacology of opioid receptor and thus their responsiveness. In addition, it has been shown that a link exist between STOP protein and MOR [16,17]. Thus, STOP disruption may directly affect MOR pharmacology and thus endogenous and exogenous morphine binding and effects. In the case of STOP null mice, such disruption is correlated with a decrease of the nociceptive threshold.

Although the STOP family protein seems to be a predictable genetic factor that may be implicated in schizophrenia, it is difficult to extrapolate our data to this neuropathology. It is true, however, that these schizophrenic patients exhibit abnormal nociception and this is particularly problematic when they require surgery. Our data suggest a possible implication of eM to finely tune the efficiency of mu receptor ligands. A clinical study aimed measuring the eM amounts together with nociceptive thresholds in schizophrenic patients will certainly be of significant interest in the near future in order to confirm the role of endogenous morphine in this pathology.

## Conclusion

In conclusion, our data show that STOP null mice display elevated levels of endogenous morphine, as well as an increase of MORs affinity and density, an observation which is correlated with hypernociception and impaired morphine receptor ligands sensitivity. The mechanistic of eM implications in these processes have to be investigated in future studies.

## Materials and methods

### Animals

STOP null mice were generated on a 50:50 BALBc/129 SvPas background as previously described [11], with gene targeting being used to replace exon 1 of the STOP gene with a non-functional construct. Experiments were performed on 37 day-old STOP null male mice and littermates, weighing 30 ± 3 g. Animals were given free access to food and water, with a 12 h light-dark cycle at a temperature of 22 ± 2°C. All experiments were carried out in accordance with the European Community Council Directive (86/609/EEC) of November 24, 1986 and were approved by the regional ethics committee and French ministry of agriculture (license No. 67-116, to P.P.).

### Drugs

Drugs were purchased from the following sources: morphine hydrochloride (Sigma Aldrich, Saint Louis, USA), Naloxone hydrochloride (Ascent Scientific, Princeton, USA). Morphine hydrochloride and naloxone hydrochloride were dissolved in saline (NaCl 0.9%), for subcutaneous injections. The effects of morphine and naloxone on nociception were tested 20 min after the injections. [N-methyl-^3^H]-morphine was purchased from American Radiolabeled Chemicals (80 Ci/mmol St. Louis, USA).

### Nociceptive tests

All nociceptive tests were preceded by at least 5 days of habituation to handling and testing procedures, in order to obtain stable basal values.

#### Mechanical nociceptive threshold measurement

The mechanical threshold leading to nociceptive hindpaw withdrawal was determined using von Frey hairs [55]. Briefly, mice were placed in clear Plexiglas^® ^boxes (7 cm × 9 cm × 7 cm) on an elevated mesh screen for a habituation time of 15 min. Calibrated von Frey filaments (Bioseb, Chaville, France) were then applied to the plantar surface of each hindpaw in a series of ascending forces (ranging between 0.4 and 26 g). Each filament was tested five times per paw. The mechanical threshold corresponded to the force of the filament inducing three or more hindpaw nociceptive withdrawals out of five consecutive trials.

#### Thermal nociception

Thermal nociceptive tests were made using a computer-controlled dynamic hot and cold-plate (Bioseb, Vitrolles, France) following our recently-described procedure [56]. The animals were placed in a Plexiglas cylinder (10 cm diameter, 15 cm height) with a drilled cover for a habituation time of 15 min. For dynamic hot plate test (DHP), animals were placed on the plate at 30 ± 0.1°C, and the plate temperature increased up to 44°C, with a 1°C.min^-1 ^speed (r^2 ^= 1). For dynamic cold plate test (DCP), animals were placed on the plate at 20 ± 0.1°C, and the plate temperature was decreased down to 1°C, with a 1°C.min^-1 ^speed (r^2 ^= 0.99). During each degree interval, for DHP or DCP, we counted the number of jumps displayed by the mouse. A cutoff value of 30 jumps was used to remove the mice from the test (always over 43°C or below 2°C). The thermal nociceptive threshold corresponded here to the temperature of first jump for each mouse. The total number of jumps is also given for hot (from 30°C to 43°C) and cold temperature ramps (20°C to 2°C). Note that only mice exhibiting jumps in DHP/DCP tests were kept for analysis. If all mice were producing jumps during the DHP test, only a small percentage of them were having this behavior in the DCP test.

### Immunohistochemistry

The presence of endogenous morphine-like compounds in mouse brain of STOP and WT animals was first revealed *via *an immunohistochemistry approach on sagittal brain slices (global morphine presence).

#### Tissue preparation for immunohistochemistry studies

Mice were deeply anesthetized by intraperitoneal injection of 0.1 ml of a 5.6% (w/v) pentobarbital sodium solution (CEVA Santé Animale, Libourne, France) and perfused transcardially with 4% formaldehyde (EMS, Hatfield, USA) in NaCl/Pi buffer (0.9% NaCl and 25 mM sodium phosphate, pH 7.4) using a peristaltic pump. Fixative solutions were chilled, and then injected for 10 min with a peristaltic pump at a flow rate of 10 ml/min. The brain was quickly removed and incubated for 2 h at 4°C in the same fixative. Coronal and sagittal brain sections (70 μm thick) were cut with a vibratome (Leica VT 1000 S, Nanterre, France) and collected in Tris-buffered saline (TBS: 50 mM Tris-HCl, 0.9% NaCl, pH 7.4).

#### Immunostaining

Immunostaining was performed on sections free-floating in TBS as previously described [57]. Brain slices were washed in TBS and incubated for 1 h in bovine serum albumin (BSA; Sigma-Aldrich) diluted in TBS (3%, w/v) in order to saturate nonspecific immunoreactive sites. After six TBS washes of 5 min each, sections were incubated overnight with a mouse monoclonal antibodies (6D6, dilution 1:1000, Aviva System Biology, San Diego, USA) raised against morphine-like compounds (morphine, M3G, and M6G, based on supplier specifications and our own experiments) [21,23].

After incubation with the primary antibody, brain slices were washed six times with TBS (5 min) and incubated with a horseradish-conjugated specific secondary antisera (HRP-conjugated donkey anti-mouse IgG, dilution 1:400; P.A.R.I.S., Compiegne, France) for 2 h at room temperature, followed by six TBS washes (5 min).

Several controls were carried out to assess antibody specificity and nonspecific immunoreactivity. The primary antibody was omitted, and the secondary antibody was tested individually or in a mixture in the presence of tissue sections or cells. Controls for morphine immunoreactivity were carried out by incubating the antibody with morphine (2 h, 25°C, 50:1, w/w) [23] prior to immunocytochemistry experiments. Anti-morphine antibody was tested by ELISA in order to determine cross reactivity with morphine, morphine-6-glucuronide (M6G), morphine-6-glucuronide (M6G), dopamine, adrenaline, noradrenaline and norlaudanosoline/tetrahydropapaveroline, showing a specificity for morphine, M6G and M3G. No cross reactivity was found for dopamine, adrenaline, noradrenaline and norlaudanosoline/tetrahydropapaveroline [21]. In order to assess whether morphine binds to proteins nonspecifically, extracts mouse hippocampus were submitted to Western blot analysis (50 μg of RIPA-extract, SDS-PAGE 4-12% acrylamide Criterion XT precast gel 12%, sample buffer 60 mM Tris-HCl pH6.8, 2% SDS, 4 M Urea, 5% glycerol, 1% β-mercaptoethanol, 5 min at 100°C); the results show no anti-morphine antibody labeled proteins in these extracts (data not shown).

#### Light microscopy immunocytochemistry studies

Peroxidase activity was observed after 20 min of incubation in a freshly prepared solution of 4-chloro 1-naphtol (0.2 mg/ml) in TBS containing 0.006% (w/v) hydrogen peroxide. After washing with TBS, the sections were mounted in glycerol/TBS (1:1, v/v) before analysis with a Leica DMRB microscope equipped with a digital camera (Axiocam, Zeiss;objectives 10×, 20× and 40×). Brain pictures were reconstructed using the Photostich 3.1 software (Canon).

### Morphine-specific ELISA

Homogenized brain areas (cerebellum, brainstem and brain) were sonicated at 4°C (3 × 10 sec) in water. Extracts were centrifuged (30 min, 10.000 *g*, 4°C), and the supernatant containing the intracellular material was extracted with methanol (1:3, v:v final ratio). After centrifugation (15 min, 10.000 *g*, 4°C), the supernatant was dried with a SpeedVac evaporator (Thermo Fisher Scientific, Brebières, France) and then dissolved in water prior to ELISA analysis. For mouse serum analysis, aliquots of 40 μl were tested in duplicates.

The morphine-specific enzyme-linked immunosorbent assay (ELISA) kit from Immunalysis Corporation (Pomona, USA provided by AgriYork 400 Limited, Pocklington, UK) was used for the quantification of morphine present in brain tissue extracts (n = 6) [23]. The specificity of the test for morphine was confirmed by testing different amounts of dopamine, adrenaline, noradrenaline, norlaudanosoline/tetrahydropapaveroline, morphine, M6G, M3G, and codeine (0.01 to 25 ng/ml, data not shown). For all tests, morphine standards were diluted in the appropriate buffer.

### Gel electrophoresis and Western blot analysis

Proteins were separated on SDS-PAGE gradient gels (4%-12% acrylamide; Criterion XT, BioRad) and electrotransferred onto polyvinyldifluorene membranes (GE Healthcare Bioscience, Sweden) [58]. In order to immunodetect the mu receptors, 50 μg of brain RIPA-extracts from WT and STOP null mice (n = 3). The MOR receptor was detected using a goat anti-MOR1 antibody (N-20; Santa Cruz Biotechnology, USA; dilution 1:1,000) and revealed using HRP-conjugated anti-goat antisera (Jackson immunoresearch, England; dilution 1:50,000) and a Supersignal West Femto Kit (Pierce, Rockford, USA). Apparent molecular weights were evaluated by comparison with molecular weight standards (Bio-Rad). Control experiments omitting the primary antibody confirmed the specificity of the label. Quantifications of the intensity of immunoreactive bands were done using the ImageJ software Version 1.43 http://rsbweb.nih.gov

### HPLC-MS/MS Instrumentation and Analytical Conditions

Few mass spectrometry (MS) methods have been reported for the qualification and the quantification of morphine and its glucuronide in biological sample [59-61]. HILIC chromatography coupled to a triple quadrupole mass spectrometry was used to develop a method to accurately detect the presence of morphine (MOR), morphine-3-glucuronide (M3G) and morphine-6-glucuronide (M6G) in the selected reaction monitoring (SRM). Identification of the compounds was based on precursor ion, one selective fragment ions and their relative retention times.

Prior to LC-MS-MS analysis, methanol extracted samples were dissolved in 0.1% trifluoroacetic acid in H_2_O (v:v) and purified using an Äkta purifier HPLC system (GE Healthcare Bioscience) as previously described [22]. Fractions corresponding to morphine were dried with a SpeedVac evaporator prior to LC-MS-MS analysis.

LC separations were carried out with an Agilent LC 1100 binary pump, autosampler, vacuum degasser, and column oven coupled with an Agilent 6410 Triple Quad LC/MS (Agilent Technologies, Palo Alto, USA).

LC separations were carried out with an Agilent LC 1100 binary pump, autosampler, vacuum degasser, and column oven coupled with an Agilent 6410 Triple Quad LC/MS (Agilent Technologies, Palo Alto, USA). The dry sample were resolvated in 10 μl of acetonitrile 70%, vortex-mixed for 1 min and injected on an acrylamido-type column (TSKgel Amide-80, TOSOH, Tokyo) at 25°C. The solvent system consisted of 100% water, 0.15% formic acid and 5 mM ammonium acetate (NH OAc; solvent A) and 100% acetonitrile (solvent B). Elution was performed at a flow rate of 220 μl/min with a 70-40% linear gradient (solvent B) over the 8 first minutes, followed by a 80% stage (solvent B) over 2 min before the reconditioning of the column at 70% of solvent B. The system was fully controlled by MassHunter software (Agilent Technologies).

Electrospray ionization was achieved in the positive mode with the spray voltage set at 4000 V. Nitrogen was used as nebulizer gas and nebulizer pressure was set at 20 psi with a source temperature of 100°C. Desolvation gas (nitrogen) was heated to 350°C and delivered at a flow rate of 10 L/min. Qualification was performed in selected reaction monitoring (SRM) mode with the following transitions: m/z 286.2 → 165.1 for morphine. The details of the optimized SRM parameters for Morphine are shown in additional file 1.

### Receptor Binding

Binding assay to brain membranes was conducted in a 96-well format. Animals were decapitated, brains were rapidly removed and homogenized with 10 volumes (w/v) of ice-cold 0.32 M sucrose using a Potter-Elvehjem tissue grinder with a Teflon pestle. The homogenate was sonicated and then centrifuged at 4°C (10 min at 1000 *g*). The pellet was discarded and the supernatant was centrifuged at 4°C for 20 min at 20,000 *g*. The supernatant was decanted and the pellet was suspended in ice-cold 50 mM Tris-HCl buffer (pH 7.4) and sonicated. After 30 min at 4°C, the suspension was resuspended and centrifuged at 4°C for 20 min at 20,000 *g*. The resulting pellet was further suspended in Tris-HCl buffer (pH 7.4) and gently sonicated. Protein concentrations were determined by the BCA method. Proteins were stored at -70°C until used.

The binding assays were performed on membrane extract (100 μg of protein in Tris-HCl buffer, pH 7.4) from a pool of STOP mice (5 brains in the pool) or WT mice (7 brains in the pool) in the presence of a concentration of 0.1 nM to 50 nM of [^3^H]-morphine ([N-methyl-^3^H]-morphine, 80 Ci/mmol, American Radiolabeled Chemicals). Nonspecific binding was determined in the presence of 10 μM morphine. Incubations were performed at 37°C during 30 min. The binding was terminated by rapid filtration of the mixture under vacuum through Whatman GF/B filters presoaked for 30 min in Tris-HCl buffer (pH 7.4). The filters were washed three times with 0.2 ml of ice-cold Tris-HCl buffer (pH 7.4) and transferred to 5 ml vials with 3 ml scintillation liquid. Radioactivity was counted 24 h later by using a Beckman scintillation counter. All experiments were conducted in triplicate. Specific binding was determined as the difference between radioligand bound in the absence (total binding) and presence (nonspecific binding) of 10 μM morphine. The GraphPad Prism program (GraphPad-Prism, San Diego, CA), was employed, with a nonlinear curve fitting analysis, to fit the nM/mg of proteins data to a saturation binding curve.

### Statistical analysis

#### Morphine ELISA analysis

In order to assess if a difference of morphine amount exist in brain tissues, determined morphine concentrations were subjected to *post hoc *analysis using a Mann-Whitney test. (STOP, n = 10; WT, n = 13). Statistical data analysis was performed using MINITAB 13.20 (Minitab Inc.). For multiple comparisons, the significance level was adjusted using Bonferroni correction. The significant level was set at P < 0.001.

#### Morphine binding assay

These statistical processes were performed using the GraphPad statistical software. *B*_*max *_and *K*_*d *_values used for statistics were calculated from the non-linear regression analysis which provides more reliable estimations (GraphPad Prism Program). However, linear Scatchard plot regression analyses were presented for rapid visual interpretation of the data. Deviation from the model non-linear regression analysis was checked with the runs test and was never significant for all experiments. The *r*² were always better than 0.96 in all cases. Statistical comparison between experimental conditions was assessed by analysis of Student's *t-*test after the confirmation that the variance aren't significantly different. A probability level of 0.05 or smaller was used to indicate statistical significance.

#### Behavioral analysis

Data are expressed as mean ± SEM. Statistical analyses were performed with KyPlot (KyensLab, Tokyo, Japan). The Wilcoxon - Mann-Whitney U test for unpaired data was used to compare non-parametric data from the von Frey hair test or the hot/cold dynamic plate. For the thermal nociceptive score as well as for the global activity tests, Student's t-test was used. The significant level was set at p < 0.05.

## Abbreviations

DCP: dynamic cold plate test; DHP: dynamic hot plate test; ELISA: enzyme-linked immunosorbent assay; eM: endogenous morphine; ESI: electrospray ionization; MS/MS: mass spectrometry-mass spectrometry; M3G: morphine-3-glucuronide; M6G: morphine-6-glucuronide; MOR: mu opioid receptor; SRM: selected reaction monitoring; STOP: Stable tubule-only polypeptides; WT: wild type.

## Competing interests

The authors declare that they have no competing interests.

## Authors' contributions

A.C. carried out the nociceptive tests, behavioral analysis, in vivo pharmacology, performed the statistical analysis and drafted the manuscript. A.M. carried out the biochemistry analysis of endogenous morphine and drafted the manuscript. A.L. carried out the immunohistochemistry and drafted the manuscript. V.K. performed the receptor binding assay and helped to draft the manuscript. A.S. produced the mouse STOP null mice. J.C.D. participated to the immunohistochemistry study and helped to draft the manuscript. D.S., participated to the ELISA analysis and the immunohistochemistry study. F.D. performed the mass spectrometry analysis. E.B. participated to the immunhistochemistry study and helped to draft the manuscript. A.V.D. participated in the design of the study and helped to draft the manuscript. D.A. participated in the study design and coordination. A.A. produced the null STOP mice, provided the null STOP mice and helped to draft the manuscript. P.P. conceived the study, participated to the statistical analysis and helped to draft the manuscript. Y.G., conceived and supervised the study, participated to the biochemistry analysis of endogenous morphine and drafted the manuscript. All authors read and approved the final manuscript

## Supplementary Material

Additional file 1 **Optimized SRM parameters for morphine**. Parameters used for the detection of morphine. Click here for file
