# Supplementary material for: Abnormal nociception and opiate sensitivity of STOP null mice exhibiting elevated levels of the endogenous alkaloid morphine
Source: Mol Pain. 2010 Dec 20;6:96. doi: 10.1186/1744-8069-6-96 (PMC3017033; doi:10.1186/1744-8069-6-96)
Supplement: Additional file 1 — Optimized SRM parameters for morphine. Parameters used for the detection of morphine. [file 1744-8069-6-96-S1.DOC]

**Supplemental data: Details of the optimized SRM parameters for Morphine.**

| **Optimized multiple reaction monitoring (MRM) parameters** | | | | | | |
| --- | --- | --- | --- | --- | --- | --- |
|  | **Precursor ion** | **MS1 resolution** | **MS2 resolution** | **Fragmentor energy (V)** | **Collision energy (eV)** | **Product ion** |
| **Morphine** | **286.2** | **Unit** | **Wide** | **161** | **44** | **165.1** |
